# The brain in motion: How ensemble fluidity drives memory-updating and flexibility

**DOI:** 10.7554/eLife.63550

**Published:** 2020-12-29

**Authors:** William Mau, Michael E Hasselmo, Denise J Cai

**Affiliations:** 1 Neuroscience Department, Icahn School of Medicine at Mount SinaiNew YorkUnited States; 2 Center for Systems Neuroscience, Boston UniversityBostonUnited States; University of Texas at AustinUnited States; University of Texas at AustinUnited States

**Keywords:** neural ensemble, engram, memory flexibility, memory updating, memory consolidation, memory allocation

## Abstract

While memories are often thought of as flashbacks to a previous experience, they do not simply conserve veridical representations of the past but must continually integrate new information to ensure survival in dynamic environments. Therefore, ‘drift’ in neural firing patterns, typically construed as disruptive ‘instability’ or an undesirable consequence of noise, may actually be useful for updating memories. In our view, continual modifications in memory representations reconcile classical theories of stable memory traces with neural drift. Here we review how memory representations are updated through dynamic recruitment of neuronal ensembles on the basis of excitability and functional connectivity at the time of learning. Overall, we emphasize the importance of considering memories not as static entities, but instead as flexible network states that reactivate and evolve across time and experience.

## Introduction

Memories are neural patterns that guide behavior in familiar situations by preserving relevant information about the past. While this definition is simple in theory, in practice, environments are dynamic and probabilistic, leaving the brain with the difficult task of shaping memory representations to address this challenge. Dynamic environments imply that whatever is learned from a single episode may not hold true for future related experiences and should therefore be updated over time. If not, memory systems will fail to generalize to future retrieval episodes in which conditions may have changed, leading to suboptimal behaviors (Richards and Frankland, 2017). Hence, the ‘goal’ of a memory system is not to remember individual events with the greatest possible precision, but rather to continually adapt its contents in order to build more and more accurate models of the world. To do this, the brain employs ‘memory-updating’, which we define as the process of modifying existing firing patterns to support the integration of new information into previously learned memories. Consistent with this concept, studies on reconsolidation have previously described how memories can become labile during retrieval allowing for memory-updating (Dudai, 2012; Misanin et al., 1968; Nader et al., 2000) and these memory modifications continue indefinitely over an animal’s lifetime (Dudai, 2012; McKenzie and Eichenbaum, 2011; Nadel et al., 2012). While reconsolidation studies have greatly contributed to our understanding of continual learning, these studies typically rely on amnesic pharmacological agents that offer limited insight to how memory modification occurs at the neurophysiological and population level. However, recent studies utilizing approaches to observe and manipulate large-scale neuronal populations have reinvigorated the search for the flexibility of memory traces (Box 1). Indeed, as we will describe in this review, these observations support the concept of continual memory-updating and complement the findings regarding molecular mechanisms in the reconsolidation literature (Tronson and Taylor, 2007). Rather than discrete and fixed neural representations, we propose that memories are stored in flexible activation patterns that are continuously modified over experience, on the order of minutes to lifetimes.

Box 1. Large-scale neuronal population recordings for studying memory.

Rapid advances over the last several decades have enabled researchers to record the activity of large populations of neurons (usually in rodents, non-human primates, and rarely humans) and relate this activity to mnemonic processes. Multielectrode drives can be chronically or acutely inserted into the brain to measure spiking from many single units through extracellular voltage. Optical techniques such as calcium imaging can monitor fluorescence from calcium indicators expressed in hundreds of neurons simultaneously. Researchers when faced with these large neuronal populations have turned to computational analyses to correlate neural activity with ongoing behavior (for a review see Cunningham and Yu, 2014). As a basis for many of these analyses, the researcher may construct ‘population vectors’ from the neural data. Typically, these vectors will describe the activity rate of each neuron binned with respect to some other variable. For example, a common way to create population vectors from hippocampal place cells is to count spikes for each recorded neuron when the animal is traversing a (binned) spatial location. These population vectors comprise an *N ×* *S* matrix where *N* is the number of neurons, *S* is the number of spatial bins, and each element in the matrix is firing rate. From these data, a researcher could search for activity patterns that may be indicative of memory encoding and retrieval processes. Key findings from this approach have identified hippocampal spiking patterns that correlate with spatiotemporal aspects of experience (Buzsáki and Moser, 2013; Buzsáki and Tingley, 2018; O'Keefe and Dostrovsky, 1971; Pastalkova et al., 2008), reactivate during memory retrieval (Foster, 2017; Joo and Frank, 2018; Lee and Wilson, 2002) and can be causally linked to behavior (Robinson et al., 2020).

### Stability versus flexibility in long-term memory

There is consensus that, generally speaking, memories are stored in activity patterns and synaptic weights of neuronal ensembles brain-wide, and that these ensembles are reactivated when recalling the memory (Frankland et al., 2019; Gelbard-Sagiv et al., 2008; Guzowski et al., 1999; Liu et al., 2012; Reijmers et al., 2007; Wilson and McNaughton, 1993). These ensembles can persist over long timespans measured over days to weeks, making them attractive substrates for long-term memory storage (Josselyn et al., 2015; Tonegawa et al., 2015). The foundation for these ideas came from Donald Hebb, whose theories on synaptic plasticity and the stabilization of cell ensembles laid the groundwork for contemporary ideas in memory representations within neuronal networks (Hebb, 1949). For instance, demonstrations of ensemble stability were found in hippocampal place cells (pyramidal neurons that fire according to the animal’s position in space) (O’Keefe and Nadel, 1978) that were found to be stable over many weeks (Thompson and Best, 1990). Stability of neuronal ensembles was also supported by studies using localization of immediate-early gene expression in the hippocampus—exploration of two identical environments 20 min apart induced activity-dependent *Arc* expression in highly overlapping populations of CA1 neurons (Guzowski et al., 1999). In the amygdala, reactivation of a neuronal ensemble active during learning was correlated with memory recall several days later, indicating a stable neural correlate for fear memory (Reijmers et al., 2007). Based on these and related studies, modern theories suggest that dedicated populations of neurons (‘engram cells’) encode and store memories in the manner of a Hebbian cell ensemble (Hebb, 1949; Josselyn et al., 2015; Tonegawa et al., 2015).

While groundbreaking, the discovery of stable ensembles as substrates for memories is an incomplete account of how memory systems operate over the course of an animal’s lifetime. Above all, these principles do not explain how the brain can integrate new experiences with old memories. In practice, some degree of flexibility must complement persistence in the successful implementation of memory (Richards and Frankland, 2017). The ‘stability-plasticity dilemma’ describes the necessary compromise between these two opposing forces, allowing new learning to occur while preserving existing knowledge (Grossberg, 1982). Indeed, modeling studies have shown that an overly rigid neural network actually encumbers the acquisition of new information. In an inflexible network, existing knowledge can interfere with the encoding of new information (proactive interference) and is also subject to erasure during new learning (catastrophic forgetting) (Hasselmo and Wyble, 1997; McClelland et al., 1995; McCloskey and Cohen, 1989). Therefore, in addition to understanding how memories can persist in neural populations, it is equally important to understand how memory systems can overcome collisions between old and new memories. To that end, dynamic memory ensembles encapsulate how memories can be both persistent and fluid. This idea is in line with the research on reversal learning (Izquierdo et al., 2017), reconsolidation (McKenzie and Eichenbaum, 2011), schema learning (Bartlett, 1932; Gilboa and Marlatte, 2017; Tse et al., 2007), and systems consolidation (Kumaran et al., 2016; McClelland et al., 1995; Nadel et al., 2012), which all describe how previously learned behaviors can be modified to accommodate new learning. In particular, prominent theories on systems consolidation stress the importance of both persistence and flexibility—the hippocampus is often thought of as the flexible learner that trains neocortical networks to store memories long term (McClelland et al., 1995). However, neocortical networks still undergo continual modifications as the animal learns over a lifetime (Kumaran et al., 2016).

Although much is known about the flexibility of behaviors, we know much less about how the memories enabling those behaviors are updated at the neurophysiological level. While the flexibility of memory has been well appreciated in synaptic neurobiology (Holtmaat and Svoboda, 2009; Rumpel and Triesch, 2016; Ziv and Brenner, 2018) and cognitive psychology (Nadel et al., 2012), it has been largely neglected by neurophysiologists with a few recent notable exceptions (Chambers and Rumpel, 2017; Clopath et al., 2017 ; Richards and Frankland, 2017; Rule et al., 2019). We begin by highlighting recent longitudinal observations of slow fluctuations in neural activity and synaptic structure that complement the ‘stable engram’ hypothesis (Josselyn et al., 2015; Tonegawa et al., 2015). Intrinsic fluctuations that alter synaptic connectivity and cellular excitability provide an ever-present reservoir of flexibility in population activity patterns to store a memory. We propose that these slow fluctuations act like a conveyor belt that continuously supplies potential new storage sites of future memories, as suggested by previous studies on 'memory allocation' and 'memory-linking' (Cai et al., 2016; Josselyn and Frankland, 2018; Rashid et al., 2016; Yokose et al., 2017). Neurons encoding these future experiences may overlap with existing engrams, updating past memories. In the following section, we describe how ‘unstable’ ensembles are not in fact paradoxical, but instead are necessary for flexible memory and behavior. Then, we outline the steps of the memory-updating process which entails (1) a partial reactivation of a previously formed engram, (2) recruitment of neuronal populations into existing engrams based on their excitability and functional connectivity, (3) deployment of plasticity processes that modify these networks by integrating the new population, and (4) temporal coordination of neural activity within and across regions for brain-wide memory-updating.

### The benefit of dynamism: how drift aids memory flexibility

#### Representational drift: findings from neurophysiology

**Figure 1.**
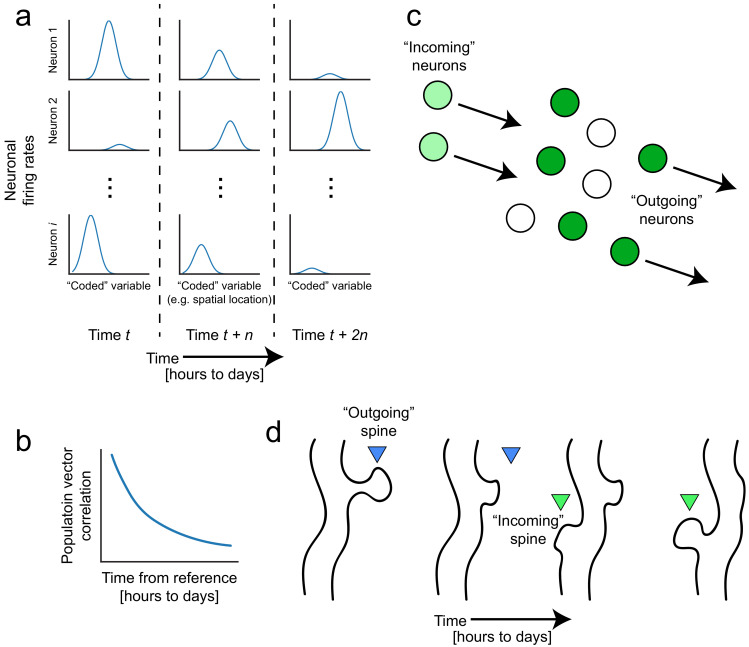
Representational drift and intrinsic dynamics supply neural substrates for memory-updating over time. (**a**) Example tuning curves of a neuronal population changing its firing patterns over time with respect to an arbitrary external variable (e.g. spatial location) (Mau et al., 2018; Ziv et al., 2013). Each column corresponds to a certain point in time. Some neurons will lose their field while others gain one. This occurs even when the animal is performing stereotyped behavior (Chambers and Rumpel, 2017; Rokni et al., 2007; Rule et al., 2019). (**b**) Schematic of population similarity over time. As a result of (**a**), the similarity of population activity to time *t* decreases over time (Mankin et al., 2012; Mau et al., 2018; Ziv et al., 2013). (**c**) Schematic of fluidity in ensembles. Intrinsic fluctuations that result in increased excitability in certain neurons (light green circles) bring them up to par with the currently active ensemble (dark green circles) relative to non-active ensembles (white circles). These ‘incoming’ neurons become more likely to encode future memories (Rogerson et al., 2014). At the same time, other ‘outgoing’ neurons lose their association with the network or their synapses are pruned to make room for the incoming cells. (**d**) Schematic of dynamic synapses. Even in the absence of neuronal activity, synapses are known to be continuously formed and eliminated (Minerbi et al., 2009; Yasumatsu et al., 2008), meaning this is an intrinsic process that occurs regardless of input. Perhaps the underlying source of drift at the population level is intrinsic synaptic volatility (Holtmaat and Svoboda, 2009; Ziv and Brenner, 2018).

With the advent of functional imaging methods that can enable longitudinal tracking of single neurons over days to weeks, a picture is starting to emerge that memory representations are not as fixed as we might expect. Despite experimenters’ efforts to keep external environments consistent and with no observed changes in the animal’s behavior, the firing patterns of neuronal populations continue to evolve, a phenomenon known as ‘representational drift’ (for reviews see Chambers and Rumpel, 2017; Clopath et al., 2017; Rule et al., 2019). Repeated exposures to the same conditions produce neural representations of these highly familiar experiences that nonetheless fluctuate on the order of hours to weeks, even with stereotypical behavior. For instance, hippocampal ensemble activity deviates over time, as measured by population vector correlations to a reference point, despite no deviations in the behavioral task (Figure 1a and b; Bladon et al., 2019; Hainmueller and Bartos, 2018; Mankin et al., 2012; Manns et al., 2007; Mau et al., 2018; Rubin et al., 2015; Ziv et al., 2013). Similar fluctuations have been reported in barrel cortex (Margolis et al., 2012), motor regions (Liberti et al., 2016; Peters et al., 2017; Rokni et al., 2007), lateral entorhinal cortex (Tsao et al., 2018), and parietal cortex (Driscoll et al., 2017), suggesting that while all these regions differentially contribute to memory, drift may be a ubiquitous feature of neural systems that seems to threaten memory stability. Alternatively, drift could actually reflect the inherent flexibility of the neural code and stem from numerous parallel neurobiological processes including spontaneous synaptic remodeling (Ziv and Brenner, 2018), and dynamic changes in cellular excitability (Figure 1c and d; Chen et al., 2020; Slomowitz et al., 2015). Next, we will consider how these processes could potentially contribute to the way in whichdynamic neural codes can support memory flexibility.

### Synaptic turnover: contributions to representational drift

Synapses are constantly being remodeled, which could be one underlying factor for drift in ensemble codes (Buzsáki, 2010; Rumpel and Triesch, 2016; Ziv and Brenner, 2018). While neural activity is a well-known mediator of synaptic plasticity (Bi and Poo, 1998; Bliss and Collingridge, 1993), the reverse relationship is also possible—synaptic structure and weights could influence neural activity patterns (Buzsáki, 2010; Fauth et al., 2015; Kalle Kossio et al., 2020; McKenzie et al., 2019; Turrigiano and Nelson, 2004). Along those lines, when action potentials are blocked in cell cultures, synapses continue to turn over across days, demonstrating that spontaneous synaptic remodeling occurs even in the absence of neuronal firing, and that these intrinsic remodeling events could impact the participation of individual neurons within ensembles (Minerbi et al., 2009; Yasumatsu et al., 2008). Hippocampal dendritic spines have been found to completely turn over within weeks, presumably contributing to fluctuations in neuronal firing patterns via rearrangement of synaptic weights (Attardo et al., 2015; Pfeiffer et al., 2018). Supporting this possibility, one experiment described how local activity patterns at CA1 dendrites predicted longitudinal place field stability based on somatic calcium activity. The experimenters found that place cells were more likely to lose their field if the dendritic activity of those cells was more variable on a trial-by-trial basis (Sheffield and Dombeck, 2015). This suggests that the computations occurring in dendritic compartments could be disturbed by synaptic turnover, which in turn could potentially destabilize place fields across the whole population. Recent modeling efforts have shown that synaptic turnover can give rise to drifting ensembles (Kalle Kossio et al., 2020). However, direct evidence for synaptic turnover as a major source for representational drift has not yet been shown and would require technically challenging feats of simultaneously imaging spines and somatic activity over long timescales.

### Stable memories from tenacious networks

While seemingly disruptive, there is substantial evidence that constant flux does not preclude memory persistence. Although dendritic spines are routinely formed and eliminated, a significant proportion is estimated to persist over an animal’s lifetime (Yang et al., 2009), suggesting that long-term memories could be stored in the synapses located at those spines. These persistent spines are more common in the neocortex than in the hippocampus (Attardo et al., 2015), consistent with systems consolidation theories suggesting that neocortical networks play a larger role in storing relatively more stable representations. Nonetheless, up to 15–25% of hippocampal CA1 neurons retain the same spatial firing patterns across weeks, which is sufficient for accurate spatial decoding (Shuman et al., 2020; Ziv et al., 2013). Such patterns may persist in part due to the relatively high resilience of larger dendritic spines (Holtmaat and Svoboda, 2009; Holtmaat et al., 2005), which has been observed in both hippocampal (Pfeiffer et al., 2018) and neocortical spines (de Vivo et al., 2017). Additionally, modeling work has suggested that repeated offline reactivation of specific ensembles could maintain potentiated synaptic weights (Fauth and van Rossum, 2019). Thus, long-term memories may be supported by a ‘backbone’ of stable spines and neurons that store gross features while the remainder might continually undergo plasticity to encode more detailed representations (Buzsáki and Mizuseki, 2014; Grosmark and Buzsaki, 2016; Sweeney and Clopath, 2020). A complementary ‘memory indexing’ theory has proposed that hippocampal neurons reinstate neocortical activity patterns for memory retrieval (Goode et al., 2020; Tanaka et al., 2018; Teyler and DiScenna, 1986). In such a regime, neocortical storage sites may house relatively more stable memories that hippocampal computations incrementally modify (Kumaran et al., 2016).

Even with the stability of a ‘backbone’ neural network, a nontrivial portion of neural networks is dynamic. Still, this does not appear to hinder memory or behavior. Performance on a variety of spatial navigation tasks remained stable across days despite continuous reorganization of firing patterns in parietal cortex and hippocampus (Driscoll et al., 2017; Kinsky et al., 2020; Levy et al., 2020). Most surprisingly, motor patterns are unchanged despite drift in neural activity from motor areas (Liberti et al., 2016; Rokni et al., 2007, but see Katlowitz et al., 2018). Perhaps behavioral stability could be attributed to the consistency of the overall population regardless of the activity of individual neurons (Rule et al., 2020). Others have shown that from a population standpoint, the variability of any individual neuron is inconsequential to the fidelity of the overall neural code (Gallego et al., 2020; Gonzalez et al., 2019; Rokni et al., 2007; Rule et al., 2019; Rule et al., 2020). A recent study proposed that downstream readers can hypothetically compensate for drift, suggesting that as long as the interpreter of an upstream neural code is capable of re-weighting its inputs, a stable readout can still be achieved (Kalle Kossio et al., 2020; Rule et al., 2020). Although the neurobiological principles governing this re-weighting have yet to be determined, they may hinge upon neuromodulatory feedback signals such as dopamine or acetylcholine (Duszkiewicz et al., 2019; Hasselmo, 2006). Taken together, these studies indicate that memory retrieval can tolerate some degree of dynamism provided that some core backbone of the collective network remains intact or if downstream readers adjust their outputs in accordance with their fluctuating inputs.

### Drift as a mechanism for continuous remodeling

What is the function of drift in memory systems? We posit that drift can slowly and stochastically provide neural substrates that can bind new information (Rumpel and Triesch, 2016), both for forming new memories and for updating old ones. In order to form new memories or update past memories, neural networks are faced with the formidable problem of having to potentiate the appropriate synaptic patterns among the huge number of possibilities that make up the synaptic connectivity space. Structural synaptic turnover through continuous spine degradation and formation could facilitate future learning by maximizing sampling across this synaptic connectivity space (Frank et al., 2018; Holtmaat and Svoboda, 2009; Kappel et al., 2015; Minerbi et al., 2009; Rumpel and Triesch, 2016; Xu et al., 2009), increasing the likelihood of achieving certain spiking patterns, and ultimately potentiating their corresponding synaptic weights. Consistent with this logic, spine turnover is critical for birdsong acquisition, fear conditioning, and spatial navigation in zebra finches and mice (Castello-Waldow et al., 2020; Frank et al., 2018; Roberts et al., 2010). Frank et al., 2018 measured spine turnover in the mouse retrosplenial cortex and found that turnover rates, even before fear conditioning and spatial exploration, positively correlated with individual ability to learn each memory. In other words, high spine turnover rates provided a greater number of new spines available for memory encoding but may have also enabled faster sampling across synaptic space and therefore a quicker arrival to a synaptic connectivity pattern that adequately encoded the new information (Castello-Waldow et al., 2020; Frank et al., 2018; Rumpel and Triesch, 2016; Xu et al., 2009). Changes in synaptic connectivity could also heterogeneously influence the likelihood of spiking (intrinsic excitability) in neuronal subpopulations, which would in turn increase their likelihood of participating in future memory-encoding ensembles (i.e., memory allocation; Box 2; Buzsáki, 2010; Chen et al., 2020; Rogerson et al., 2014; Yiu et al., 2014; Zhou et al., 2009). In this way, the brain could prioritize different rosters of neurons to diversify eligibility for memory encoding or updating (Margolis et al., 2012; Rogerson et al., 2014; Trouche et al., 2016). Such a framework is consistent with systems consolidation where information is constantly redistributed across the neocortical-hippocampal loop (Kumaran et al., 2016; McClelland et al., 1995). In summary, spontaneous synaptic remodeling can supply additional synapses and neurons in which memories can be laid down as they are being experienced, contributing to memory flexibility.

Box 2. Neuronal excitability and memory allocation.

The memory allocation hypothesis states that neurons with high excitability are more likely to be recruited for memory encoding (Rogerson et al., 2014). Experimental excitation of subpopulations of amygdala neurons biases fear memory storage to those cells, and ablation of that subpopulation abolishes the memory while activation of that subpopulation induces memory retrieval (Han et al., 2007; Rogerson et al., 2016; Yiu et al., 2014; Zhou et al., 2009) In CA1, place cells (as opposed to non-place cells) exhibit electrophysiological properties indicative of high excitability such as lower spiking thresholds (Epsztein et al., 2011) and artificial excitation endows non-place cells with place fields (Bittner et al., 2015; Diamantaki et al., 2018; Lee et al., 2012). Also in CA1, *c-fos*-positive populations that distinguish between spatial contexts similarly show high mean firing rates (Tanaka et al., 2018). In other brain regions such as prefrontal cortex and nucleus accumbens, highly excitable cells have been found to be critical for reproduction of certain behaviors such as conditioned freezing and reward seeking (Whitaker et al., 2017; Ziminski et al., 2017), demonstrating their role in stable memory encoding and retrieval. In humans, excitability during pre-encoding also appears to predict subsequent memory encoding strength (Urgolites et al., 2020).

### Neurogenesis and plasticity aid memory-updating

Drift may also facilitate the updating of previously learned memories by partially weakening old activity patterns in favor of strengthening new ones. In particular, hippocampal adult neurogenesis has been shown to play an important role in synaptic and circuit remodeling, supporting memory-updating and flexibility (Aimone et al., 2009; Burghardt et al., 2012; Denny et al., 2014; Epp et al., 2016; Frankland et al., 2013; Rangel et al., 2014; Richards and Frankland, 2017; Saxe et al., 2006). The addition of newborn neurons in the dentate gyrus reconfigures existing hippocampal activity by decaying previously potentiated synapses (Alam et al., 2018; Frankland et al., 2013; Kitamura et al., 2009). At first glance, this may appear disruptive for memory, but integration of these newborn neurons into hippocampal circuits can be useful because network-wide synaptic reorganization can erase obsolete patterns while making room for new ones (Alam et al., 2018; Frankland et al., 2013; Richards and Frankland, 2017; Toni et al., 2008). This feature is especially advantageous for memory-updating, which requires circuit reconfiguration in order for previous memories to accommodate potentially conflicting new information. In alignment with this hypothesis, exercise and environmental enrichment (which both increase neurogenesis) enhanced navigational flexibility when a goal was relocated (Epp et al., 2016; Garthe et al., 2016). In these studies, mice with increased neurogenesis were better able to learn by modifying their expectations of where the goal was likely to be, but this ability was abolished when neurogenesis was inhibited (Burghardt et al., 2012; Epp et al., 2016; Garthe et al., 2016). Importantly, neurogenesis was not required for initial memory acquisition, but only when the mice had to reverse their behavior (Burghardt et al., 2012; Epp et al., 2016). This suggests that neurogenesis plays an important role in memory-updating, and not just in acquisition of a new memory (Meshi et al., 2006). Taken together, these findings suggest that after integration of adult-born neurons, the consequent synaptic reorganization (Toni et al., 2008) allows experienced brains to acquire yet more information to integrate with previously learned associations.

Aside from neurogenesis, spontaneous synaptic remodeling is a critical feature for permitting memory-updating through the exploration of synaptic connectivity space using prior knowledge as a starting point. Modeling studies have demonstrated that continual reconfiguration of synaptic weights is necessary for a network to adapt once dynamic conditions are imposed (Ajemian et al., 2013; Duffy et al., 2019; Kappel et al., 2018). One particular study used a neural network to perform a simulated motor task where specific outputs were rewarded (Kappel et al., 2018). The authors then changed the output of a subset of model neurons relative to the motor task, thereby perturbing the neural network’s ability to produce the correct motor actions. However, because the neural network was able to continuously explore alternative solutions through a drift-like synaptic rewiring mechanism, it quickly adapted to the perturbation and regained high accuracy (Kappel et al., 2018). This suggests that slow synaptic turnover may facilitate the ability to draw from a prior knowledge base (by storing connectivity patterns that are slow to decay) while still flexibly exploring related options through stochastic probing of new potential connectivity patterns, built atop existing ones. Such an implementation may underlie flexible behaviors that are based on memories for past outcomes. In other words, as long as a memory is retrievable and the ensemble encoding that memory is sufficiently plastic, it can be updated; but suppressing retrieval (Yokose et al., 2017) or plasticity (Guo et al., 2018) blocks memory-updating. The notion of memory modification after retrieval has also been previously discussed in the context of reconsolidation, when memories are subject to modification after they are retrieved (Dudai, 2012; McKenzie and Eichenbaum, 2011; Misanin et al., 1968; Nader et al., 2000). In the next section, we examine how the brain modifies these memories.

### Neural mechanisms of memory-updating

#### Memory retrieval is associated with a re-visitation of neural state space

There is a large body of evidence showing that memories are not created de novo and instead draw upon prior knowledge stored in biophysical configurations of synapses and population activity (Bliss and Collingridge, 1993; Dragoi and Tonegawa, 2014). As such, memory-updating relies on instantiating an internal representation of past events, comparing expectations to current sensory input, and modifying the representation to improve predictive power (Gershman et al., 2017). For example, in humans, viewing specific images reactivates the firing patterns observed during initial presentation of the stimulus (Gelbard-Sagiv et al., 2008). In rodents, similar neuronal populations are reactivated upon re-exposures to familiar environments (Guzowski et al., 1999; Reijmers et al., 2007; Rubin et al., 2015; Shuman et al., 2020; Thompson and Best, 1990; Ziv et al., 2013), and activation of neuronal populations previously activated during the initial learning will induce memory retrieval (Liu et al., 2012; Ramirez et al., 2013; Robinson et al., 2020).

**Figure 2.**
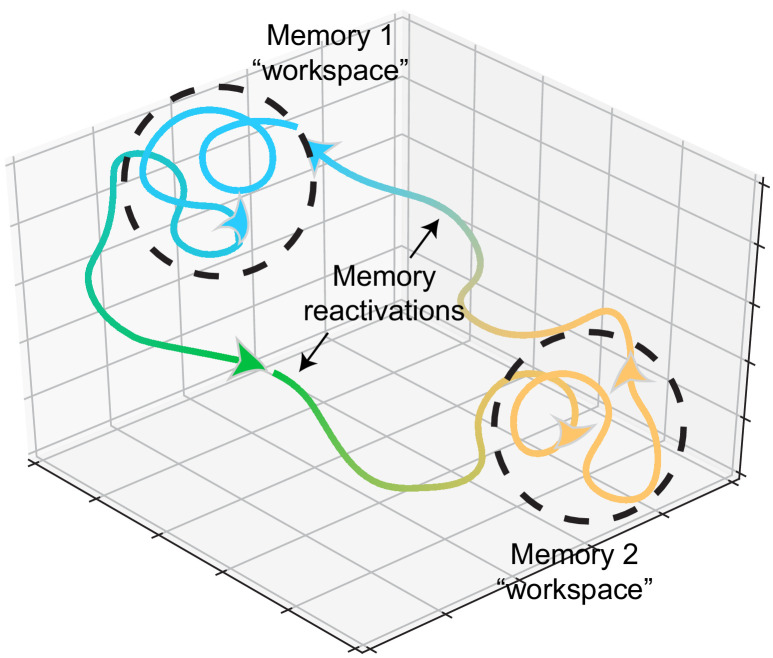
Memory representations occupy regions of state space during experience and learning. Example trajectories of network states during recollection of two memories, depicted on a neural subspace. The network state is expressed in the firing activity of large neuronal populations. During situations where the environment or context is relatively stationary, the network state exhibits slow drift that constrains learning and plasticity locally (dotted black circles). Upon a major contextual shift, the network state responds with a fast, commensurate shift to a new regime (from Memory 1 to Memory 2, green trajectory) that recalls another memory. The network resides there until another contextual shift kicks it back to Memory 1. In experimental conditions, these contextual shifts are usually experimenter-defined (e.g., placing animals in different enclosures). However, in the wild, they may be shaped by major changes in the animal’s surroundings. In humans, and probably in non-human animals as well, contextual shifts may be internally motivated (i.e., spontaneous recall). Compartmentalization in network state space ensures that learning does not corrupt existing memories while allowing mechanisms for memory modification within local state space regions.

Reactivation of neural patterns during memory retrieval may constrain the brain to these state spaces, restricting the degrees of freedom for exploring possible solutions for new encoding. These states may act as continuous attractors (Leutgeb et al., 2005; Tsodyks, 1999; Wills et al., 2005) and function as workspaces for memory-updating within that time window (Figure 2). Such a mechanism would imply that learning something associated with a previously learned stimulus would reactivate that previous memory. This view is again consistent with reconsolidation experiments where a reminder cue reinstates a previous memory, creating an opportunity for modification of that memory (Gisquet-Verrier et al., 2015; Hupbach et al., 2007; McKenzie and Eichenbaum, 2011; Nadel et al., 2012). Functional imaging experiments in the human brain found that presentations of reminders triggered reactivation of hippocampal and prefrontal networks, which facilitated learning of new, related items (Schlichting and Preston, 2014; Zeithamova et al., 2012). Rodent studies have also found reactivation of hippocampal ensembles during memory-updating (Dupret et al., 2010; McKenzie et al., 2013; McKenzie et al., 2014). Previous training on memory tasks with one set of stimuli accelerated their ability to form new associations with different stimuli. Upon introduction of these new stimuli, neurons that encoded previous, related stimuli were reactivated, suggesting that this population acted as a scaffold upon which new, related stimuli were embedded (McKenzie et al., 2013; McKenzie et al., 2014). This reactivation facilitates the connection of related experiences across time, allowing for the integration of events through the co-activation of neurons encoding past and present experiences. Such a mechanism is consistent with reports of optogenetic stimulation to co-activate ensembles to update previous memories with experimenter-defined ‘false memories’ (Ohkawa et al., 2015; Ramirez et al., 2013; Vetere et al., 2019).

### Memory-updating through temporal integration

To determine which experiences get integrated into a past memory, the relevance of input patterns might be weighted by temporal proximity to when the memory was encoded—events that occur close in time can become linked within neural networks (Clewett et al., 2019; Howard et al., 2014; Rogerson et al., 2014; Schlichting and Preston, 2014; Yetton et al., 2019). A functional imaging study in humans found that over two encoding episodes, subjects were more likely to combine their memories of overlapping items (object pairs) if they occurred within 30 min of each other (Zeithamova and Preston, 2017). Within encoding episodes (defined by minutes-long temporal blocks), there is high similarity in hippocampal activity patterns in both humans (Ezzyat and Davachi, 2014; Hsieh et al., 2014) and rats (Bulkin et al., 2020), suggesting that the network resides in the same state space during these episodes. Likewise in mice, two contextual experiences within 5 hr of each other became represented by a common set of hippocampal CA1 neurons, but not when they were separated by 7 days (Cai et al., 2016). Furthermore, not only were those two experiences represented by an overlapping ensemble, but when one of those contexts was subsequently paired with shock, animals transferred the fear from the shocked context to the neutral context. This indicates that the two memories became linked, which was sufficient to update a recent neutral memory with an aversive association (Cai et al., 2016). In another study, mice were fear conditioned with two different tones, separated by 6 hr, and these two tone-shock associations were represented in the amygdala by a common set of neurons (Rashid et al., 2016). When one of the tones was repeatedly presented without shock (a paradigm known to lead to extinction of freezing), it also reduced freezing for the other tone, suggesting that the two memories became linked within those 6 hr. These effects were not seen when longer time intervals separated the two encoding events. Yet another study was able to link a conditioned taste aversion to a fear conditioned response through an overlapping population encoding the two conditioned stimuli (Yokose et al., 2017). These ‘temporal memory-linking’ studies demonstrate that events occurring in close temporal proximity can mutually impact and modify memory representations of surrounding events.

### Excitability and synaptic dynamics ensure a constant supply of neurons for memory-updating

During memory-updating, how does the network determine which neurons are integrated into an existing memory? Accumulating evidence indicates that excitability and functional connectivity influence the allocation of neurons into memory ensembles. This theory, known as the ‘memory allocation hypothesis’, suggests that the excitability of a neuron predisposes it for encoding a memory (Box 2; Rogerson et al., 2014; Zhou et al., 2009). Moreover, the excitability and connectivity of neuronal populations are constantly changing, which would suggest that different sets of neurons encode or update memories as these neurons increase in prominence from the perspective of the network (Buzsáki, 2010; Chen et al., 2020; Minerbi et al., 2009; Slomowitz et al., 2015). In other words, drift, as a result of constantly fluctuating excitability (Chen et al., 2020; Rogerson et al., 2014; Slomowitz et al., 2015), synapses (Holtmaat and Svoboda, 2009; Rumpel and Triesch, 2016; Xu et al., 2009; Yang et al., 2009; Ziv and Brenner, 2018), and intracellular proteomes (Rogerson et al., 2014), define the degree to which single neurons compete to participate in encoding a memory (Han et al., 2007) based on their excitability and connectivity to an existing memory engram.

During memory-updating, in order to homeostatically maintain ensemble sizes (Stefanelli et al., 2016), other neurons must also decrease their roles in memory encoding, ‘exiting’ the ensemble by reducing their contribution to the memory representation (Figure 1c). Because synaptic potentiation is saturated in engram neurons (Choi et al., 2018), a neuron’s exit from an ensemble might reflect depotentiation, reducing its likelihood of being co-activated with the remainder of the ensemble and potentially allowing it to encode other information. Depotentiation could result from a new competing ensemble suppressing the activity of neurons from a previous ensemble (Han et al., 2007; Rashid et al., 2016). For example, ensembles formed during extinction may be inhibiting ensembles formed during fear learning (Lacagnina et al., 2019). As secondary evidence, optogenetic inhibition of an engram results in recruitment of an alternative engram (Rashid et al., 2016; Schoenenberger et al., 2016; Trouche et al., 2016), implying that the ensemble that might have been ‘next in line’ to compete with the first acts as a fail-safe to encode the present experience, ensuring functional homeostasis. Such competition may also underlie the loss of firing selectivity (e.g., receptive fields) over time in some neurons, decreasing their contribution to reliable memory encoding of the original memory (Liberti et al., 2016; Mankin et al., 2012; Manns et al., 2007; Mau et al., 2018; Rubin et al., 2015; Sheffield and Dombeck, 2015; Trachtenberg et al., 2002; Ziv et al., 2013). The gradual departure of those neurons may allow a transition to another set of neurons that surface with new information to integrate into the existing memory.

During memory-updating, memory allocation on the basis of neuronal excitability promotes the overlap between ensembles encoding memories close in time. High excitability biases neurons encoding one memory to be recruited to encode another event, thereby linking the two memories through a shared neuronal ensemble (‘allocate-to-link’ hypothesis) (Cai et al., 2016; Rashid et al., 2016; Rogerson et al., 2014). But how could associations be made between experiences that are more temporally distant? One possibility is through ensemble reactivation during memory retrieval, which prepares an old ensemble to integrate new ensembles (Yokose et al., 2017). Because memory retrieval involves a reactivation of a neuronal ensemble, excitation of those cells could trigger a physiological state that primes them for integrating the activity patterns of new neurons into their network. In line with this idea, learning increases neuronal excitability (Chandra and Barkai, 2018; Disterhoft et al., 1986). Early studies found that eyeblink conditioning (Disterhoft et al., 1986) and operant conditioning (Saar et al., 1998) increased neuronal excitability in CA1 and piriform cortex by decreasing the magnitude of slow after-hyperpolarizations (due to decreases in the calcium-dependent hyperpolarization current that follows spike bursts). Additionally, recent experiments have shown that during encoding and retrieval episodes, excitability is elevated in specific populations—the engram cells (Cai et al., 2016; Pignatelli et al., 2019). By reactivating these engram cells during memory retrieval, they become re-excited along with newly excited cells at the next encoding event, which could induce synaptic potentiation between these ensembles (Abdou et al., 2018; Choi et al., 2018; Nabavi et al., 2014). In doing so, this would combine memories that are related in content (yet encoded distant in time) in a common set of neurons. Simply put, the brain could recruit neurons encoding new information to fire alongside engram cells into an ensemble that now incorporates elements of new and old firing patterns (Ohkawa et al., 2015). This modified ensemble then represents the updated memory.

### Pre-existing temporal motifs influence neuronal recruitment patterns

**Figure 3.**
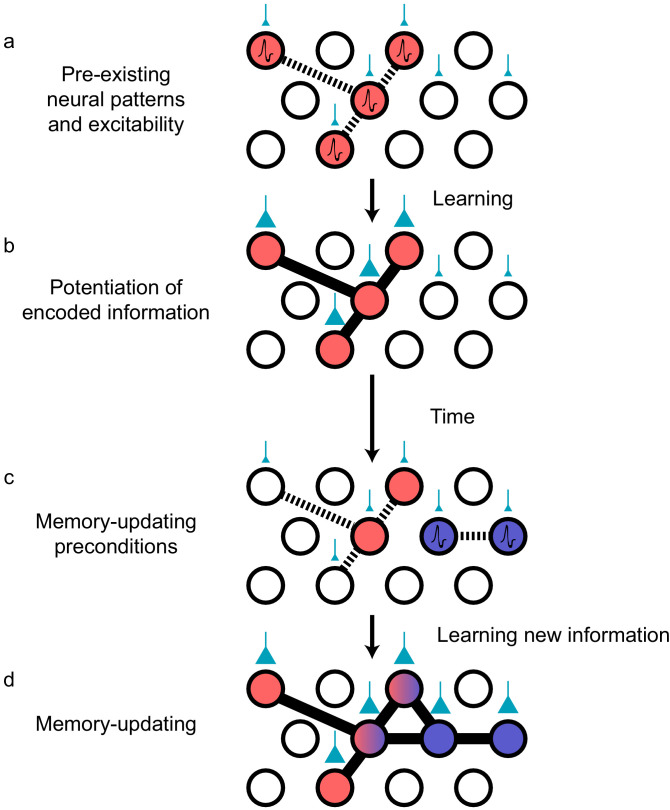
Preconfigured synaptic weights and excitability bias the allocation of memories to certain neuronal populations. (**a**) Prior to an encoding event, some neurons will happen to be at the higher end of a distribution of excitability (red circles). These cells may be synaptically connected with each other, or receive shared input from upstream regions (e.g., CA1 receiving input from CA3; cyan boutons with bouton size representing synaptic weight) that result in high functional connectivity (dashed lines). Other cells may also be receiving input but have low excitability (empty circles). (**b**) During learning, these highly excitable cells increase their functional connectivity (bold lines connecting red circles) through synaptic plasticity. This could be achieved through potentiation of direct synaptic connections in the case of recurrent brain regions (e.g., CA3), or through potentiation of synapses in upstream regions that achieve appropriately timed co-activation of downstream (e.g., CA1) ensembles. (**c**) Over time, synaptic weights may weaken but some may persist to allow partial reactivation of a prior ensemble during memory recall (red circles). At the same time, just as in (**a**), a new population of cells may exhibit above-average excitability and functional connectivity (blue circles and dashed lines) at the time of a second learning episode. (**d**) Learning may potentiate functional connections between the red and blue ensembles to link the two memories.

While excitability could determine the identity of the neurons to be recruited into an ensemble during memory-updating, pre-existing firing patterns (i.e., functional connectivity) at the time of encoding could mediate the temporal structure of the new neural motif destined for eventual potentiation (Dragoi and Tonegawa, 2014; McKenzie et al., 2019). The temporal structure of spiking patterns could then be interpreted by downstream reader regions (Buzsáki and Tingley, 2018). An observed phenomenon that supports the hypothesis of pre-existing temporal patterns is hippocampal ‘preplay’ (Figure 3a). Preplay refers to an ensemble of CA1 neurons that produces reliable sequential activity *prior to spatial learning*, which is then recapitulated by firing in the same order while exploring physical space (Dragoi and Tonegawa, 2011; Dragoi and Tonegawa, 2014, but see Silva et al., 2015). This finding again implies that memory representations are not created de novo and instead that pre-existing functional connections and premature synaptic patterns can become potentiated by experience (Figure 3b). That is, there is always likely to be a neuronal sequence that occurs more often than others by virtue of the current state of synaptic connectivity (Buzsáki and Mizuseki, 2014), and this sequence becomes most likely to encode the next upcoming memory. In this way, the current state of the network as it drifts is connected to ongoing experience through the storage of information in neuronal ensembles with precise temporal coordination.

Neural sequences derived from preplay templates may undergo cyclical refinement, consistent with our views on continuous memory-updating and how pre-existing temporal structure can shape the patterns that emerge. Once a preplay pattern is potentiated, increasing its occurrence rate, it is considered to be ‘replay’ (Dragoi, 2020; Foster, 2017; Liu et al., 2019) and it is likely that replay patterns could then take on the role of preplay templates to integrate upcoming, related events (Dragoi, 2020). Modification of these sequence templates would therefore be an efficient mechanism for flexibly learning new information that is consistent with pre-established networks, without the need for widespread restructuring (McClelland et al., 2020). In support of this idea, pre-existing sequences are relatively preserved after learning but reorganization is still apparent; new neurons are added to a backbone sequence after spatial exploration (Grosmark and Buzsaki, 2016; Figure 3c). One study investigated the properties of neurons that begin to co-fire with an established sequence after spatial exploration of a novel environment (Grosmark and Buzsaki, 2016). This study found that newly recruited neurons contained higher spatial information, suggesting that the neurons that are most informative about spatial regularities in the environment are preferentially added to specific positions along the sequence. Moreover, consistent with the memory allocation theory, the neurons that were recruited tended to have higher firing rates than those that were not (Fernández-Ruiz et al., 2019; Grosmark and Buzsaki, 2016). Finally, the recruitment of neurons is dependent on the degree of temporal coordination between those neurons both prior to and during experience, demonstrating that functional connectivity among the ensemble participants is critical for recruitment (Farooq and Dragoi, 2019; Farooq et al., 2019). Thus, both excitability and functional connectivity bias the composition and temporal coordination of recruited neurons during memory-updating. But to actually induce physical changes in the synaptic strengths of these ensembles, the brain must modify synapses using plasticity proteins in concert with temporally coincident neural activity.

### Deployment of plasticity proteins enables subsequent synaptic potentiation of new ensembles

How do slowly drifting synaptic configurations and spiking patterns actually stabilize to store information? One major factor is the upregulation of experience-dependent plasticity, which can temporarily stabilize memory ensembles. Experience-dependent plasticity can then potentiate activity patterns of memory ensembles to allow for those patterns to be retrieved for the next time they are to be updated. Indeed, there is enhanced expression of plasticity-related proteins such as cAMP responsive element binding protein (CREB) in numerous brain regions following memory retrieval and updating (Hall et al., 2001). Artificial memory retrieval, through activation of engram cells, triggers plasticity in those cells and memory modification at the behavioral level. For instance, after fear-conditioning, chemogenetically activating CREB-expressing amygdala engram neurons induces freezing and also triggers reconsolidation-associated protein signaling cascades that may prime memory-updating (Kim et al., 2014). Blocking plasticity with protein synthesis inhibition prevents memory-updating and impairs learning-associated changes in firing patterns and synaptic turnover (Dragoi and Tonegawa, 2013; Dupret et al., 2010; Kim et al., 2014; Li et al., 2017; Tse et al., 2011). The myriad protein signal pathways triggered by learning also diversifyneuronal electrophysiological properties, such as excitation and inhibition patterns. These signaling cascades may enable complex interplay among a heterogeneous population to support and balance different aspects of memory, such as stability and flexibility (Sweis et al., 2020; Yap and Greenberg, 2018). For example, a recent study found that *Fos*- and *Npas4*-expressing granule cell ensembles in the dentate gyrus were respectively responsible for generalization and discrimination of contextual fear memory (Sun et al., 2020). Further study is required to fully understand how constellations of molecular components interact in the cellular and network milieu to support high order cognition.

### Temporal coordination of neuronal ensembles promotes neuronal recruitment and memory-updating

In addition to the mobilization of plasticity mechanisms, neurons also need to spike in a temporally coordinated fashion in order to be recruited (Bi and Poo, 1998). Once neurons become coordinated at the millisecond scale, the resultant ensembles can then persist over longer timescales owing to synaptic potentiation (Bi and Poo, 1998; Bliss and Collingridge, 1993). Oscillatory patterns in the brain represent an organizational framework for grouping the spike timing of neuronal subpopulations, which has led to a large body of literature on how rhythms could subserve experience-dependent plasticity, and therefore also memory-updating. In the hippocampus, plasticity is thought to be mediated by temporally coordinated neuronal ensembles within brief time windows such as the sharp-wave ripple (SPW-R) envelope (Buzsáki, 2015) and individual cycles of the 4–12 Hz theta rhythm (Colgin, 2013; Hasselmo et al., 2002; Larson et al., 1986). Both theta oscillations and SPW-Rs are powerful instigators of plasticity and long-term potentiation (LTP). In vitro, theta-paced stimulation is the optimal frequency for inducing LTP in hippocampal slices (Larson et al., 1986). Importantly, the theta phase is critical for determining whether LTP or synaptic depression occurs (Hyman et al., 2003), meaning that not only is the magnitude of theta important, but also the spike timing of neuronal ensembles within theta cycles (Colgin, 2013; Dragoi and Buzsáki, 2006). Recent studies have shown that CA1 neuronal spiking needs to be temporally organized within theta cycles in order to be recruited into SPW-R events after learning (Chenani et al., 2019; Dragoi, 2020; Drieu et al., 2018; Farooq and Dragoi, 2019; Farooq et al., 2019). Thus, theta oscillations corral ensembles for potentiation and memory-updating.

After temporal coordination, SPW-Rs may accelerate the rate of plasticity among the newly recruited neurons during memory-updating to integrate them into the ensemble. Artificial induction of SPW-Rs strengthens Schaffer collateral synapses in vitro (Sadowski et al., 2016), but curiously also causes long-term depression in synapses that were *not* recently active (Norimoto et al., 2018). This result suggests that recently activated ensembles are preferentially strengthened while connectivity with less relevant neurons is eroded, resulting in enhanced signal-to-noise. Consistent with this view, hippocampal SPW-Rs refine and stabilize place field maps in animals learning novel environments (Dupret et al., 2010; Gridchyn et al., 2020; Roux et al., 2017; van de Ven et al., 2016). This process is facilitated by upregulating SPW-R prevalence during learning. As a rat learns a spatial memory task, SPW-Rs increase in duration, and optogenetically prolonging SPW-Rs increases behavioral performance and recruits neurons that have spatial fields in behaviorally relevant locations (Fernández-Ruiz et al., 2019). This preferential recruitment implies a mechanism for prioritizing integration of neurons that encode pertinent information and consequently serve important functional roles (Kinsky et al., 2020; Michon et al., 2019). SPW-Rs likely bind old and new memories by co-activating the appropriate ensembles. The end product is an updated ensemble whose spiking patterns convey updated information about recently learned stimuli (Dupret et al., 2010; Fernández-Ruiz et al., 2019; Grosmark and Buzsaki, 2016; O'Neill et al., 2008; Roux et al., 2017; van de Ven et al., 2016).

### Sharp-wave ripples coordinate cross-regional memory-updating

Hippocampal SPW-Rs could also recruit extrahippocampal neurons, which might help transmit and integrate recent hippocampal computations with knowledge stored in the neocortex to facilitate brain-wide memory-updating (Kumaran et al., 2016; McClelland et al., 1995). The reorganization of neocortical spiking patterns could then readjust local (cortical) synaptic weights in a way that promotes meaningful activation patterns, synthesizing updated memories that may support schemas (Benchenane et al., 2010; Gilboa and Marlatte, 2017; Kumaran et al., 2016; Maingret et al., 2016; Rothschild et al., 2017). This cross-regional dialogue is a signature of systems consolidation, where the hippocampus is thought to train neocortical ensembles by developing and reinforcing population patterns over a lifetime of experiences (Kumaran et al., 2016; McClelland et al., 1995). Indeed, widespread activation of cortical regions is time-locked to hippocampal SPW-Rs (Logothetis et al., 2012), and SPW-R coupling between neocortex and hippocampus becomes upregulated after learning (Khodagholy et al., 2017). After learning, hippocampal SPW-Rs trigger spiking in downstream cortical ensembles, and also couple with cortical oscillatory events such as ripples, spindles, and delta waves (Alexander et al., 2018; Benchenane et al., 2010; Khodagholy et al., 2017; Latchoumane et al., 2017; Maingret et al., 2016; Rothschild et al., 2017; Todorova and Zugaro, 2019). These neocortical oscillatory events might themselves facilitate local plasticity. Although the individual functions of each frequency band remain an active area of research, generally, cross-regional coherence triggers spike pattern reorganization in downstream readers of hippocampal SPW-Rs that could be the neurophysiological readout of memory-updating (Benchenane et al., 2010; Kumaran et al., 2016; Maingret et al., 2016; Rothschild et al., 2017). Recent evidence suggests that SPW-Rs can route specific content, suggesting that different memories can be individually communicated to downstream readers and updated separately (Gridchyn et al., 2020). Hippocampal sequences, and their downstream neo- and subcortical readers, could reflect a modified ensemble structure that balances both parsimony and newly acquired information after undergoing experience-dependent plasticity.

## Concluding remarks

In this review, we have discussed forms of memory-updating and their neurophysiological signatures. We have proposed that these processes can be understood through the modification of neuronal ensembles whose membership is determined by both existing circuitry between neurons and intrinsicdynamics within neurons. Our framework is based on the idea that the integration of ensemble activity creates functional affinities (i.e., related memories). The organization of the neurons within these ensembles is heavily influenced by a combination of cellular excitability and functional connectivity (pre-existing temporal activity patterns) and the enactment of plasticity that modifies synaptic weights. While substantial work has shown that ‘stable’ engram neurons underlie memory retrieval (Josselyn et al., 2015; Liu et al., 2012; Tonegawa et al., 2015), they do not necessarily persist indefinitely without modification. Instead, to satisfy the stability–plasticity dilemma, ensemble activities across brain regions must necessarily be fluid in order to update past memories with new information.

While our review discusses how engrams change over time and experience, there is much still unknown about how the brain resolves the end products of memory-updating. How do these new modifications reconcile with established patterns? Previous studies have found that learning induces enduring changes : population activity does not revert to its original state when a familiar rule is reintroduced (Dupret et al., 2010; Grewe et al., 2017; Malagon-Vina et al., 2018; McKenzie et al., 2013). These findings suggest that interleaved learning episodes can have lasting effects on the computational outputs of mnemonic systems, reflecting acquisition of new knowledge (Kumaran et al., 2016). One particularly interesting idea is that this new knowledge might be integrated with the old via the recombination of sub-ensembles, facilitating the formation of new relational topologies (Sweis et al., 2020; Ghandour et al., 2019).

An interesting effect of dynamic ensembles is that ‘tuning’ from neuronal populations relative to the external world deteriorates over time, which explains many observations of apparent neural instability (Chambers and Rumpel, 2017; Driscoll et al., 2017; Mankin et al., 2012; Mau et al., 2018; Rubin et al., 2015; Rule et al., 2019; Ziv et al., 2013). That is, the patterns of neuronal responses related to external stimuli are short-lived. Instead, the internal mappings *between* neurons persist through time. From the reference frame of the environment, the neural code for some variable (e.g., spatial location) may be drifting, but from the reference frame of the neurons, representations might remain stable due to compensatory adaptation at the network level (Kalle Kossio et al., 2020; Gonzalez et al., 2019; Kinsky et al., 2018; Rule et al., 2019; Rule et al., 2020). Such an outcome may arise from slow, coherent representational shifts such that different brain regions collectively shift in a coordinated manner. Among these regions may be ‘readers’ that interpret the messages of memory ensembles, and if those networks remodel coherently with upstream inputs, this could support the continuity of memories over time (Buzsáki, 2010; Kalle Kossio et al., 2020; Rule et al., 2020) while also providing a means to update those memories. Thus, it is important to consider how inter-cellular activity patterns are modified during learning, and one major future avenue of research should be to determine how memory-updating rearranges internal activity patterns in an organized manner that retains past information while integrating new data. Most importantly, this perspective forces us to re-assess our very notions of stability. Nearly all central nervous system neurons are many synapses away from direct contact with physical stimuli, so if the upstream populations that do supply their input are inherently dynamic, we should rarely expect a neuron to maintain a stable relationship to an external variable even in the context of memory. Instead, we take the view that brain dynamics help generate unique combinatorial patterns onto which both new and familiar experiences are embedded.

While many outstanding questions persist (Box 3), emerging technologies will prove critical for enhancing our understanding of the dynamic brain. In particular, voltage imaging (Adam et al., 2019; Piatkevich et al., 2019) has the advantage of being able to track neurons longitudinally while also providing exquisite temporal resolution and sensitivity to signals that are undetectable with calcium imaging (e.g., subthreshold depolarizations). Combining this with other optical techniques in freely behaving animals (Cai et al., 2016; Ziv et al., 2013) will allow us to advance our understanding of the dynamic brain.

Box 3. Outstanding questions.

- What is the neurobiological milieu that determines the rate of representational drift? Do neuromodulators, sleep, or neuroendocrine signals exert influence?
- Are drift rates in brain regions related to their local intrinsic synaptic volatility (Attardo et al., 2015)? What are the consequences of these differences and how might this relate to the rate of memory-updating in hippocampal versus neocortical structures (Kumaran et al., 2016)?
- How does ensemble remodeling occur in order to retain stored information while adding new information?
- Downstream of memory representations, what are the circuits and computations that transform these firing patterns into behavioral decisions?
